# EFFECTS OF SPECIAL NURSING UNITS IN NURSING HOMES: FOCUSING ON HEALTH OUTCOMES AND SATISFACTION

**DOI:** 10.1093/geroni/igac059.2034

**Published:** 2022-12-20

**Authors:** Eunhee Cho, YeaSeul Yoon, Seonhwa Choi, Eunkyo Kim

**Affiliations:** Yonsei University, Seoul, Seoul-t'ukpyolsi, Republic of Korea; Yonsei University, Seoul, Seoul-t'ukpyolsi, Republic of Korea; Yonsei University, Seoul, Seoul-t'ukpyolsi, Republic of Korea; Yonsei University, Seoul, Seoul-t'ukpyolsi, Republic of Korea

## Abstract

This quasi-experimental study aimed to evaluate the effectiveness of special nursing units in nursing homes by comparing health outcomes and satisfaction between the general nursing units and special nursing units across nursing homes in South Korea. Surveys—paper, phone, and online—were conducted five times (March, August, and November 2019; June and October 2020) for health outcomes (health pattern changes and the number of residents with new health problems) and four times (March and August–November 2019; June and October 2020) for satisfaction. Descriptive analysis, χ²-test, paired t-test, and McNemar test were performed using the SPSS 25.0 program. The results showed an improvement in the health outcomes of residents in the special nursing units. Regarding health pattern changes, there was a decrease in the number of residents facing problems related to consciousness, cardiovascular function, urination, defecation, and pain and of those with new health problems such as aches, falls, pressure sores, and urinary incontinence. Furthermore, caregivers’ satisfaction in the special nursing units was higher than that of their counterparts in the general nursing units. The results of this study will be used as evidence to expand this special nursing unit’s services in the future.

